# Exploring mental health clients' current medication knowledge, beliefs and experience with healthcare providers in the community in South Australia

**DOI:** 10.1111/hsc.14029

**Published:** 2022-09-23

**Authors:** Tien Ngoc Thi Bui, Elizabeth Hotham, Mark Loughhead, Sara S. McMillan, Nicholas Procter, Kessie Poole, Vijayaprakash Suppiah

**Affiliations:** ^1^ UniSA Clinical and Health Sciences University of South Australia Adelaide South Australia Australia; ^2^ Mental Health and Suicide Prevention Research Group University of South Australia Adelaide South Australia Australia; ^3^ Quality Use of Medicines Network, Menzies Health Institute Queensland Griffith University Gold Coast Queensland Australia; ^4^ Sub‐Acute Services Mind Australia Limited Adelaide South Australia Australia; ^5^ Australian Centre for Precision Health University of South Australia Adelaide South Australia Australia

**Keywords:** community health, medication counselling, medication knowledge, mental disorders, mental health, shared decision making, therapeutic relationship

## Abstract

In Australia, mental illness has been recognised as a National Health Priority area, with the coronavirus pandemic adding a layer of urgency to the need to address the multiple health problems faced by clients with mental illnesses. Whilst much has been done in efforts to support these clients, little is known about their medication knowledge and experience with health professionals. The aim of the study was to explore the knowledge and beliefs of clients on the use of psychotropic medications and study their experiences with healthcare providers. Adult participants at a not‐for‐profit community‐managed specialist mental health service provider in Adelaide, South Australia were recruited. Four focus group sessions were conducted between February 2020 and March 2021. All sessions were co‐facilitated by a peer practitioner with lived experience. Sessions were audio recorded and transcribed verbatim. Participants (*n* = 27) reported that provision of medication education was inadequate and, in some cases, non‐existent. There was an apparent lack of support for monitoring and managing common side effects, such as weight gain. Participants described not being involved in any decision‐making processes and that establishing and maintaining a therapeutic relationship with their healthcare providers was challenging. Perceived stigma remains a barrier in accessing healthcare. Despite participants regularly interacting with a range of healthcare providers, findings highlight key gaps in care, particularly medication education and establishing a therapeutic relationship with their healthcare providers. Future mental health reforms should consider the provision of additional medication education in community settings, such as at not‐for‐profit organisations. Moreover, healthcare providers should take a proactive approach in establishing therapeutic relationships.

**What is known about this topic?**

- Individuals diagnosed with mental illness often report high rates of psychotropic medication discontinuation.
- Medication taking behaviour is influenced by a client's medication knowledge, attitudes and beliefs.
- Having a positive therapeutic relationship between clients and treating healthcare providers can positively influence medication taking behaviours.

**What the paper adds?**

- Clients with mental illness receive inadequate medication education of their psychotropic medications which is further compounded by challenges in establishing and maintaining an ongoing therapeutic relationship with their healthcare providers.
- Monitoring of adverse effects proved to be the most challenging aspect of pharmacotherapy for clients with mental illnesses living independently in the community.
- Shared decision making was not immediately evident in this study.

## 1 INTRODUCTION

Mental illness is a leading cause of ill‐health and disability worldwide, affecting more than 970 million people globally (James et al., 2018). In Australia, nearly half the population aged between 16 and 85 years will experience a mental illness at some stage in their lifetime (Australian Institute of Health and Welfare, 2020). The coronavirus (COVID‐19) pandemic has further exacerbated health problems for clients with mental illnesses (Galletly, 2020). Recent mental health reforms in Australia have seen the delivery of services transform from previously being inpatient‐reliant to a more community‐based service (Australian Government Department of Health, 2009). This transition, combined with existing schemes such as Medicare (which covers all costs associated with services provided in public hospitals, general practitioners and medical specialists) and the Pharmaceutical Benefits Scheme (PBS) (with the provision of all PBS listed medicines at a subsidised price), aimed to make healthcare more accessible and affordable for Australians, especially for individuals diagnosed with mental illness. Although much work has been done in reforming the health system, more effort is required, especially in supporting positive medication taking behaviours in individuals with mental illness (e.g. improving medication adherence).

The majority of individuals diagnosed with mental illness (hereon, simply referred to as ‘clients’) are often prescribed psychotropic medications. Studies on the effectiveness of these medicines are often related to the concept of treatment adherence, despite noting high rates of discontinuation in clients living with schizophrenia (20%–72%) and major depressive disorder (28%–52%) (Julius et al., 2009). Medication discontinuation is concerning, given the significant correlation between medication discontinuation and re‐hospitalisation (Green, 1988; Kozma & Weiden, 2009), as well as higher rates of relapse and suicidal behaviour (Clatworthy et al., 2007; Colom et al., 2005).

Medication taking behaviour is influenced by clients' medication knowledge, attitudes and beliefs. In particular, positive medication beliefs can play a major role in medication taking behaviour (Drivenes et al., 2020; Higashi et al., 2013), with medication beliefs being influenced by knowledge of medication effects (Grover et al., 2014). Similarly, people with greater knowledge of the therapeutic effects of medications have also been shown to be more likely to have a positive attitude towards their medications (Nagai et al., 2020; Wiesjahn et al., 2014). In addition to enhancing medication knowledge, establishing a positive therapeutic relationship between clients and their healthcare providers can also positively influence medication taking behaviours (Day et al., 2005).

Despite the recognised importance of having adequate medication knowledge, research indicates that education provided by doctors and pharmacists may be inadequate (Kessler, 1991; Tully et al., 2011; van Dijk et al., 2016). An Australian study found that medication education was insufficient and clients' (*n* = 9) experience with healthcare providers was unsatisfactory (Happell et al., 2004). For example, Young et al. (2006) found that doctors do not regularly provide important information, such as anticipated duration of therapy and expected delay in onset of action to people commencing antidepressants. Furthermore, reports have shown that up to 60% of clients rarely or never receive written medicine information (Knox et al., 2015).

The present study aims to explore community clients' (i) knowledge and beliefs on the use of psychotropic medications and (ii) their experiences with their healthcare providers. This will enable health professionals, especially those working in community settings to identify current gaps in care and work with clients to positively influence their medication taking behaviours.

## 2 METHODS

### 2.1 Design

This qualitative study recruited adult participants at a not‐for‐profit (NFP) community‐managed specialist mental health service provider in Adelaide, South Australia. In order to maximise participant numbers, facilitate open discussion and allow for the exploration of a wide range of experiences, face‐to‐face focus groups were employed. The delivery of the sessions via a virtual platform was not feasible as most of the clients did not have access to the electronic means required. As this study was done during the COVID‐19 pandemic, the sample size was further impacted by limitations to public gatherings imposed by the South Australian government. The ideal sample size for each focus group was set between 6 and 10 participants (Powell & Single, 1996). The study was approved by the institution's Human Ethics Committee (202299) and the mental health service provider's ethics committee.

### 2.2 Recruitment

Peer practitioners (PP) employed at the NFP were briefed on the study and led the recruitment phase. Participants were approached face‐to‐face and recruitment flyers were displayed in the main waiting area at the two NFP locations. Participants were encouraged to discuss with the PP if they were interested or had any questions about the study. Convenience sampling was chosen as the recruitment strategy. Study inclusion criteria consisted of: (i) clients who have or are currently taking medication for any mental illness, (ii) aged 18 years or older and (iii) able to give informed consent.

### 2.3 Procedure

Prior to the focus groups, study information and consent forms were provided and explained to participants, with an opportunity to ask questions prior to undertaking the focus group. Written consent was obtained from all participants prior to commencing the focus groups. The sessions were facilitated by researchers (M.L. and T.B.), who conducted two focus groups each. M.L., a lived experience researcher is an academic with extensive experience in qualitative research. Researcher T.B. is a registered pharmacist and PhD candidate with research interests in mental health and qualitative methods. Both researchers had no established relationship with any of the participants prior to the study's commencement. The researchers' background (e.g. research interests, work experience and whether they have lived experience) were also highlighted to the participants prior to the commencement of the focus group sessions. All focus groups were co‐facilitated by a PP with lived experience (Mental Health Coalition of South Australia, 2020). Researchers VS and EH were present at the sessions as silent observers and EH made field notes during the sessions. The sessions were informed by a discussion guide which had been previously piloted with a lived experience academic and a lived experience end user (Appendix S1). The four focus groups were held in meeting rooms at two NFP centres between February 2020 and March 2021 and sessions ran for an average of 54 minutes. The study followed the grounded theory approach (Chun Tie et al., 2019; Walker & Myrick, 2006), with two focus groups being conducted in 2020 and data analysed prior to additional focus groups being conducted in 2021. An honorarium of AUD $30.00 gift voucher was provided as an appreciation for the participants' time.

### 2.4 Data analysis

The focus groups were audio‐recorded and transcribed verbatim by the researcher (TB). Transcripts were not returned to participants for comments but were available when requested. Data were analysed using an inductive approach (Thomas, 2006). Thematic analysis was conducted by researcher TB and analysis was guided by the six‐step method discussed by Braun and colleagues (Braun & Clarke, 2006). To enable data familiarisation, the researcher re‐visited the audio recordings and reviewed transcripts several times. An initial list of codes was drawn from the first review of transcripts. Transcript texts were then manually coded and identified codes were matched with data extracts using an Excel® spreadsheet. Codes were collated into potential themes, then reviewed and compared with initial list of codes and field notes to ensure that they accurately represented the data. Findings (themes and subthemes) were also reviewed and discussed by researchers (T.B., V.S. and E.H.) and made available to the PP who was present at the focus group sessions to establish an accurate representation of the data. To ensure the trustworthiness of the data, transcription was checked independently by two researchers (V.S. and E.H.). Any disagreements were then discussed between the researchers (T.B., V.S. and E.H.) until consensus was reached.

Data saturation was assessed using the inductive thematic saturation approach (Saunders et al., 2018), and was deemed as reached when there was no emergence of new codes or themes (Saunders et al., 2018). Researchers (T.B., V.S. and E.H.) also noted that a significant level of data saturation had been achieved by the fourth focus group, similar to findings by Guest and colleagues who suggested that 90% of all themes were discoverable within as few as three focus groups (Guest et al., 2017).

### 2.5 Reflexivity

Researcher's individual background, experience and prior assumptions can have an impact on the process of data collection and interpretation. In order to minimise this, active reflexivity was employed throughout the study (Dodgson, 2019). In particular, during the processes of study design, data collection and analysis.

In designing the study, the decision to conduct the sessions at a NFP centre where the participants frequently attend and view as a familiar place was consciously made. In doing so, we hoped for participants to view the researchers as guests in the setting, allowing for participants to feel that they are exercising control over the session. In addition, the study consisted of a diverse team that included academics with and without lived experience, PP, practicing and non‐practicing pharmacists.

During data analysis, the themes were reviewed by all researchers present at the sessions, including the silent observers (E.H. and V.S.) and the PP. This was to ensure that the perspective of one group of participants was never over‐presented and to minimise any researcher bias.

## 3 RESULTS

### 3.1 Demographics

A total of 27 participants attended the four focus groups (Table 1). One participant withdrew from the study prior to the commencement of the session for unspecified reasons. Over half of the participants were female (55.6%), 37% aged between 41 and 50 years and most had been accessing services at the mental health service provider for more than 18 months (55.6%). The majority (66.7%) of participants reported that they were currently on prescription medication(s) with antipsychotics being the most prescribed psychotropic agent.

**TABLE 1:** Participant demographics (*n* = 27)

	*N* (%)
Age (years)	
18–24	1 (3.7)
25–30	2 (7.4)
31–40	9 (33.3)
41–50	10 (37.0)
51–60	3 (11.1)
61–70	2 (7.4)
Gender	
M	12 (44.4)
F	15 (55.6)
Length of time attending the community‐managed specialist mental health service provider	
<3 months	5 (18.5)
3–6 months	1 (3.7)
6 ‐ 12 months	4 (14.8)
12–18 months	1 (3.7)
>18 months	15 (55.6)
Unspecified	1 (3.7)
Currently on medications	
Yes	18 (66.7)
No	4 (14.8)
Not specified	5 (18.5)

Psychotropic medications	Frequency
Antipsychotics	41
Antidepressants	14
Lithium	6
Benzodiazepines	2
Others	6

**FIGURE 1:**
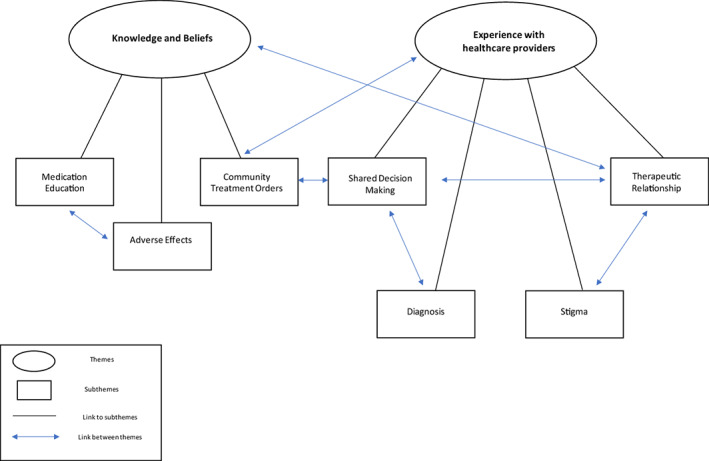
Thematic map. Visual presentation of themes and subthemes identified and relationships between categories

Participants' responses to the focus groups were categorised into the following themes and subthemes (Figure 1): (1) Knowledge and beliefs towards psychotropic medications (including medication education, psychotropic medication: adverse effects and Community Treatment Orders) and, (2) Experience with healthcare provider (including shared decision making, diagnosis, therapeutic relationships and stigma).

### 3.2 Theme 1: Knowledge and beliefs towards psychotropic medications

#### 3.2.1 Subtheme 1: Medication education

Majority of the participants did not feel confident in their knowledge of prescribed medications for their mental illness (Table 2). Only one participant was the recipient of what they believed to be substantial medication education, recalling:

**TABLE 2:** Themes and subthemes with illustrative quotes extracted from focus groups

Theme	Illustrative quotes
Theme 1: Knowledge and beliefs towards psychotropic medications	
Subtheme 1: medication education	‘my doctor did not give me any information (FG1‐P11).’ ‘I was just given things in hospital I think I was on some sort of order so I had to take whatever I was given but I did not really know what I was given if that makes sense (FG4‐H).’ ‘Once you are out of hospital it is [referring to the provision of medication education]. When you are in hospital they say oh you'll be taking this, but they do not say why or what it's for (FG3‐P4).’ ‘I think that in the time that I went on different medicines I was in manic episodes so if they explained anything to me, I probably would not even remember (FG2‐P2).’ ‘I've never been given any print outs or anything from the pharmacist at all (FG3‐M)’ ‘You find your own resources (FG3‐P6).’ ‘… normally the pharmacists will ask if you have taken it before or whatever or they'll check for any interactions and then they'll go and actually print it out and then give it to you (FG3‐A).’
Subtheme 2: psychotropic medications (side effects)	‘It turned me into a fat pig basically (FG1‐P1).’ ‘I put on 20 kg in about 4 months (FG1‐P10).’ ‘Oh once I gain weight with that medication a whole other load of problems and then that doctor was like oh no and then I had to go on more medications… (FG3‐A).’ ‘Yea I was a matchstick before they put me on medication (FG4‐C).’ ‘I had the same trouble with my weight. I was 63 kg when I went into institution and my highest was 150 kg. So now I've got more medications to speed up my metabolism (FG4‐D).’ ‘I had anorexia and so I was 37 kg most of my life so when they [doctors] put me onto medications like I said to them, like if you put me on one with weight gain I'll end up starving myself to death because I have a mental illness relating to weight … It was heaps traumatic cause I've been hospitalised all through my twenties with anorexia and they put you on a medication, the whole sort of thing with anorexia is control one thing you can control is your weight when they take that option away from you, you lose your mind (FG1‐P4).’ ‘… my main issue [with] the side effects … is the feeling that I'm losing my intelligence, awareness, you know, my perception of things, memory so it just so it just I do not like using this word but it's the only way I can really get it across … it just makes me feel a bit not stupid but like a zombie. Zonked out (FG3‐A).’ ‘*…* it's my level of awareness I cannot drive (FG4‐H).*’* ‘I need counselling [referring to medication induced emotional bluntness from Lithium] … I do not know how to deal with this life that I've got now… I do not know how to deal with the nothingness (FG2‐P2).**’**
Subtheme 3: Community Treatment Orders	‘I was chucked on this medication against my will (FG1‐P3).’ ‘I was held down and they injected me, and I was overdosed, it nearly killed me (FG1‐P4).’
Theme 2: Experience with healthcare provider	
Subtheme 1: shared decision making	‘I'm not an assertive guy and I find that I be assertive … and it gets me nowhere, so I learn to not be assertive (FG3‐P4).’ ‘… when you try to explain the story to someone else, they are like no nah you have got these problems. You know, like yea it's a tricky situation (FG4‐G).’ ‘… I want to discuss changing my medication and she [psychiatrist] does not want to hear it. She's just … No. She does not want to know and if anything, she decides maybe we just add another one (FG3‐P6.)’ ‘… because I said that I was a bit worried about continuing to gain weight and everything and I've got high cholesterol, diabetes everything that goes with it and he goes that they are just the things that you have to accept um when you are trying to stay well. And I felt like, I did not feel listened to at all. It felt like oh you are just a second‐class citizen you just have to deal with all these side effects we do not bother too much about it (FG4‐H).’
Subtheme 2: diagnosis	‘There's just a label and they look at it without dissecting what they are doing. It's more of just an overview, oh yeah, his got this symptom his got that symptom oh yes this. Oh, it's not his got that factor his got this factor so that factor (FG3‐P4).’ ‘They're more trying to cover it up, just like patch you up, keep you coming back … (FG4‐G).’ ‘They do not look at different factors. Like I've been in the system for 20 years … They did not even believe my story of trauma when I entered the system so I'm getting medicated on their thoughts not on, not on justified information so it takes another 10 years to justify yourself to them and you have been medicated and not, not supported with your trauma so you do not get the right therapy (FG3‐P4).’
Subtheme 3: stigma	**‘**I do not know if that's the case in [all] pharmacies, but I have difficulty with just instantly being judged. You know they will see my name and ask for extra information [e.g. driver's licence etc](FG3‐ A).**’** ‘I think also depends on where you are in socioeconomic [status] …[for example] cops said to me, you have no credibility cause you are a drug addict and you have mental health issues and so it's like no respect whatsoever. They do not think they have to explain anything, they just like try to shut you up (FG1‐P5).’

‘When I was diagnosed years and years ago … she [doctor] gave me some options … I get access to her [doctor] to be able to ask questions … and printed information as well … I was certainly one of those people that had had information relay [sic] to me on a number of occasions plus I had a really supportive husband who was also fully aware of all the implications and side effects of the medications (FG1‐P10).’

Other participants disclosed that they were given no information by their healthcare providers, with one participant stating, ‘I discovered more with my mobile phone these days than what I was told for the last 20 odd years (FG2‐P2).’

Some participants could not recall whether medication counselling was provided as they were acutely unwell at the time and could not process detailed information. Medications administered in the hospital were similarly not explained.

‘In the emergency room they didn't say anything they just gave me the injection. I didn't know what it is, it must have been like to knock me out or whatever. Fair enough. No, they didn't say what it was or anything (FG1‐P5).’

Participants expressed that medication education should be provided repeatedly stating:

I just think that if people are really unwell when they commence on the medication so they're not in a position to actually understand what it is that they are going to be taking. I think that … after a couple of weeks or after they start improving, people should then take the time to sit down and go over the medications even though the people are already taking them … just down the track a bit it would be nice if the doctor took the time to explain everything (FG4‐H).

and,

‘I had to be educated every single time … I find sometimes it's not the first time you hear it, sometimes second time or third time [for it to make sense] (FG1‐P10).’

#### 3.2.2 Subtheme 2: Psychotropic medications (adverse effects)

Most participants expressed concerns with the lack of counselling for potential medication‐related adverse effects. The majority of the participants claimed that they were made aware of adverse effects only after they had occurred. Moreover, it was identified that most participants did not receive counselling and/or monitoring for common adverse effects for long‐term medications. A participant felt that the current healthcare system was ill‐equipped to provide adequate aftercare care for clients such as regular monitoring of side effects.

Among the adverse effects identified, weight gain and effects on mental alertness were the most commonly discussed (Table 2). The impact of weight gain was reported to not only impact physical health, but also described as having a significant impact on their mental health:

I ended up with a whole eating disorder just to manage the weight gain with that. So it really needs to be tailored … prescribing a medication for someone with eating disorder and it's known for gaining weight, you should monitor them more clearly cause you're gonna end up with a whole other problem that will require a whole other treatment and medication…(FG3‐A).

The negative impact on mental alertness was also highlighted as something that has significantly affected their quality of life.

… the medication made me feel defenceless. I can't even protect myself (FG3‐X).

Despite the concerns previously identified, the vast majority of participants indicated that they accepted the role of prescribed medication(s) for their conditions.

I know to keep on with the medications, ‘cause it keeps me not sort of [in] a happy place but it's better than what the alternative is (FG2‐P1).

#### 3.2.3 Subtheme 3: Community treatment orders

Participants who were under a Community Treatment Order (CTO) reported very different experiences compared to those who were not. In general, participants who reported a more positive experience with the healthcare system were not under a CTO, had better rapport with their healthcare providers and had stable family and/or peer support. They also appeared to be more proactive in their treatment and had a more positive attitude towards their medications. For example, a participant (FG1‐P10) mentioned:

I researched finding a GP [general practitioner] through people's recommendation because I am fussy.

and

‘I'm aware of the fact that, something like having the family involved or carer [is important] so I asked the psychiatrist if I can have a meeting with my family on the subject of my medications …’

Alternatively, participants under the CTO were more likely to have had a negative experience with their healthcare providers, and highlighted reduced autonomy:

The autonomy is completely stripped away. It's just to do as you're told like a child, and we're not children (FG3‐P6).

These participants felt disrespected and not in control of their treatment. Whilst they understood what a CTO entailed, they felt that there should have been more aftercare. For example, one participant described:

You're too unwell to really consent to treatment but you need the medication. So, you might be under the order or something like that, but then in order to continue taking that it would be nice if someone sat down and didn't assume that because you're on [the medications] you've consented and you're really happy to continue taking this for how many years (FG4‐H).

### 3.3 Theme 2: Experience with healthcare providers

#### 3.3.1 Subtheme 1: Shared decision making

Nearly all of the participants described that they were not involved in any decision making regarding their therapy. They voiced that their concerns and opinions were not listened to by healthcare providers:

We get angry because we're not being heard and then we get angrier because the pharmacists or doctors are reacting like they're surprised that someone with a mental illness is being angry (FG3‐A).

One participant described healthcare providers over‐riding her opinions:

So when you want to explain yourself to them like, this is why I do this, this is why I won't do that, you can completely be … No. They won't take you into consideration. I've lived with this all my life so I know what's going on there and they come in and disregard everything you have to say ‘cause [sic] they're the specialist on you (FG3‐P6).

In addition, a number of participants felt coerced into their treatment therapy. For example:

Well I was asked if I would like to go onto clozapine cause they thought it was the best thing for me and they said that, that was the quickest route out of [name of psychiatric facility] was to go on clozapine. So they kinda rail‐roaded me into it (FG1‐T).

#### 3.3.2 Subtheme 2: Diagnosis

Most participants felt that the healthcare providers, when making their diagnosis, did not consider other factors, such as underlying trauma. The healthcare system was perceived as providing a greater emphasis on pharmacological treatment rather than psychological support:

… I wasn't schizophrenic like they had diagnosed me when I was in hospital I was just traumatised from my friend taking his life and I didn't know how to handle it (FG3‐A).

and,

Well emotions cause reaction, and these are some things that they [healthcare providers] don't weigh up I guess. They sit there and focus purely on symptoms not on what's caused the symptoms (FG3‐P4).

#### 3.3.3 Subtheme 3: Therapeutic relationships

It was expressed that healthcare providers should take a proactive approach in establishing and nurturing the healthcare provider–patient relationship. In particular, the constant rotation of doctors in public hospitals and community care teams was identified as a barrier to establishing therapeutic relationships. A participant expressed:

That's what sucks about the public system as well, it's because they [doctors] change over just when you get [to know them] … He was good [last psychiatrist], what if the next one doesn't have the same opinion as him? … Like he tells what the plan is and the plan can change when you get a new psychiatrist (FG1‐P4).

Some participants highlighted that whilst their experiences with treating doctors had not been ideal in the past, they have since found doctors with whom they have a good therapeutic relationship with:

They [certain GPs] don't really want to know about it, the mental side of it, whereas the GP I've got now is wonderful (FG2‐P1).

and,

I feel very confident with the GP I've got now and I feel that if I ask her any questions, I feel that she'll answer and if I want to know anything she'll explain (FG2‐P2).

#### 3.3.4 Subtheme 4: Stigma

The perception of stigma remained a barrier for clients in accessing quality healthcare. Visits to a pharmacy were described by one participant as ‘anxiety inducing (FG3‐A)’. Most participants also highlighted their struggle with finding and retaining a suitable GP for their long‐term mental health support. This then led to being perceived by pharmacists in some instances as a form of ‘doctor shopping’ (Sansone & Sansone, 2012). It was revealed:

… it looks like we're doctor shopping or whatever … this ties back to that individual care and analysing of that client ‘cause you need to look into their situation, has this person changed doctors? Are they in an environment where they can't [get] access to the same healthcare professionals (FG3‐A)?

In addition, a number of participants felt that they were treated differently based on their socioeconomic status. In response to a participant who claimed to have had an overwhelmingly positive experience in her treatment so far, a participant stated ‘… you're well respected … versus someone on a DSP [Disability Support Pension] and you know with past drug addiction (FG1‐P4).’ Participants also reported a difference in how their care was delivered when they attended their medical appointments with a companion, such as with a family member or carer:

I've noticed a difference when doctors give me injection when [a] carer [is] with me and when they're not… When [name] comes in with me they are a lot more careful about how they give me my injection. Now that I'm going in by myself [to medical appointments] they keep jabbing in me and keep getting it wrong (FG1‐P5).

## 4 DISCUSSION

The study was able to explore clients' current knowledge and beliefs towards psychotropic medications and identified opportunities for healthcare providers to further support clients in the community. The findings indicate a low level of confidence in medication knowledge among participants. For example, clients reported not being aware of common psychotropic adverse effects until they have experience it themselves, suggesting inadequate medication education. The study also identified an absence of established therapeutic relationship attributed to the lack of continuity in providers (e.g. the regular rotation of doctors) and shared decision making in this cohort. Despite this, the participants expressed an overall acceptance and understanding of the role that their psychotropic medications have for their mental illness.

Inadequate medication education among clients diagnosed with mental illnesses remains a concern (Fejzic et al., 2017; Happell et al., 2004). This situation has been previously identified as a ‘major source of dissatisfaction’ for clients (Happell et al., 2004). In particular, clients were dissatisfied with the lack of medication counselling on common adverse effects of psychotropic medications, such as weight gain and effects on mental alertness (Covell et al., 2007; Morrison et al., 2015). Given the prevalence of these adverse effects, adequate counselling and monitoring should be in place in order to reduce harm and assist clients' continuity with treatment (Roughead et al., 2017). Additionally, it was apparent that majority of the participants did not receive any form of written information and often resorted to seeking out information on the internet, as highlighted by others (Roughead et al., 2017; Stomski & Morrison, 2018). Future initiatives should endeavour to address this disparity, especially given the known association between medication knowledge and medication adherence (Nagai et al., 2020; Wiesjahn et al., 2014). Currently, medical practices and pharmacies are the most common places for medication counselling (Pohjanoksa‐Mäntylä et al., 2011). With growingly demanding work environments in medical practices and pharmacies, as seen with the global coronavirus pandemic (Johnston et al., 2021; Marshall, 2021), providing additional medication education at an alternative community setting such as NFP centres by trained health professionals may be a potential solution.

It was also apparent that perceived stigma and ‘othering’ (i.e. the view of ‘Us’ and ‘Them’) (MacCallum, 2002) remains a barrier for clients seeking quality healthcare. We propose that therapeutic relationships should be employed as a basis for addressing issues of stigma and promoting opportunities for shared decision making (SDM) which could also facilitate successful uptake of medication education, shared monitoring and review. It is paramount that all mental healthcare providers recognise that therapeutic relationship is fundamental to care (Priebe & Mccabe, 2008). In particular, healthcare providers should take the initiative in forming a trusting and professional relationship with their clients (Verhaeghe & Bracke, 2011).

We found a lack of evidence to suggest that SDM existed in this cohort. SDM is defined as the ‘approach where clinicians and patients make decisions together using the best available evidence’ (Elwyn et al., 2010). It should be recognised that majority of clients are able to make adequate decisions about their care and therefore should be central to decision‐making processes regarding their treatment (Calcedo‐Barba et al., 2020), including decisions to withdraw from medicines. Involving clients in the decision making is empowering as it recognises their expertise in their illness, respects their autonomy and promotes patient engagement (Alguera‐Lara et al., 2017; Elwyn et al., 2010; Slade, 2017), all of which have been shown to lead to better outcomes (such as better use of medicines, reducing errors and stigma) (Dixon et al., 2016; Slade, 2017). Unsurprisingly, the lack of SDM is particularly evident for clients who were under a CTO (Brophy et al., 2019). A part of the South Australian Mental Health Act 2009, the CTO stipulates that ‘treatment may be given despite the absence or refusal of consent to the treatment’ (Government of South Australia, 2018). It is important to recognise that clients on a CTO are also likely to be seen as being more unwell (Government of South Australia, 2018), but still perceive the CTO as being controlling, coercive and disrespectful (Corring et al., 2017). The study draws attention to the importance of validation and counselling support from a trauma‐informed approach, especially for clients who have experienced harm or trauma from involuntary treatment (Sweeney et al., 2018). To empower clients to participate in SDM, effort should be made in supporting clients to develop the necessary knowledge and skills to make these informed decisions (i.e. self‐management) (Schulman‐Green et al., 2012). Applying both SDM and support for self‐management will promote the making of better and more appropriate clinical decisions that would be acceptable to both the client and the treating healthcare provider (Lewis‐Barned, 2016).

It was apparent from our findings that there is a need for better integration of a person‐centred care (PCC) approach in mental health service delivery. In our study, participants felt that assessments made by healthcare providers (e.g. when making a diagnosis) did not factor in other individualised factors such as personal trauma. PCC places a greater emphasis on communication, encouraging patients to participate in their own medical treatment by working closely with their healthcare providers, leading to better‐shared collaborations and decision‐making processes (Delaney, 2018). Hence, PCC is considered to be a guiding principle for service delivery in mental health settings (Choy‐Brown et al., 2020). As highlighted by Hamovitch and colleagues, there is a ‘bidirectional relationship between PCC and therapeutic relationships and that both areas must be fully developed in order to capitalised on their benefits (Hamovitch et al., 2018)’. It is worth noting that whilst PCC should be encouraged in practice, implementation should be made with consideration. For example, Miller and colleague stressed the need to recognise the importance of learnt intuition and skills of trained healthcare providers such as GPs in providing patient care. The authors emphasised the importance of integrating PCC to encourage a doctor‐led and patient‐centred approach rather than a patient‐led care approach (Miller & Fritz, 2019).

### 4.1 Limitations

Findings must be understood within the context of the study's strength and limitations. One major strength of the study is the strong research partnership with its inclusion of lived experience researchers. It is worth noting that the views expressed in this study represent a small sub‐section of mental health clients; therefore caution should be exercised when extrapolating findings to the greater cohort. However, it is possible that our findings may also be observed in other jurisdictions such as the United States and Canada where mental health services are increasingly being delivered within community‐based settings (Dixon et al., 2016; Drake & Latimer, 2012). In addition, the study did not collect data on treatment duration, and this may affect the interpretation of our findings. For example, the experience of someone who has been on psychotic agents for 20 years can be vastly different from someone who has only recently been initiated on a psychotropic medication. We recognise that the presence of PPs, who were familiar with the clients, during the focus groups can increase the risk of clients providing socially desirable responses to the focus group questions (Grimm, 2010). The coronavirus pandemic resulted in strict restrictions being placed on public gatherings which prevented further recruitment and conduct of additional focus groups, thereby limiting the sample size. However, the authors assessed that data saturation was reached after the fourth focus group and therefore additional sessions were unlikely to have added to the current findings. The authors acknowledge that the lack of use of a specialised qualitative software such as NVivo may be a limitation, in particular in storing and managing emerging codes and themes.

## 5 CONCLUSION AND IMPLICATIONS

The study was able to add a detailed narrative of clients' experiences with healthcare providers and identified areas that remain unaddressed. Our findings revealed significant gaps in the provision of medication education and experiences with healthcare providers that require immediate attention. The study further underlined the importance of individualised and respectful care, with an urgent need to improve therapeutic relationship which would facilitate better SDM and PCC between clients and healthcare providers.

Participants described a lack of confidence in their medication knowledge, often attributed to either not receiving adequate medication counselling or being unable to recall the information provided during the counselling. Despite this, majority of the participants accepted the role of their medications in supporting their mental health but described the significant impact of psychotropic medication adverse effects on their quality of life. Providing additional medication education at an alternative community setting by trained health professionals may be a viable long‐term solution. This would allow for repetition of information in a setting that is likely less intimidating, thus facilitating better medication understanding.

Experiences with healthcare providers indicate perceptions of stigma, absence of established therapeutic relationships and shared decision making. In particular, participants who were not on a CTO described having a better rapport with their healthcare providers, were more proactive in their treatment and had a more positive attitude towards their medications. This is pivotal especially given the influence that knowledge and attitudes towards medication can have on treatment adherence.

Future initiatives should address the variation in medication education to further support and encourage positive medication taking behaviours in clients with mental illnesses. Healthcare providers should also take a more proactive approach in establishing therapeutic relationships and providing pertinent medication education, on a regular and ongoing basis.

## AUTHOR CONTRIBUTIONS

The authors VS, EH, NP and ML contributed to the study conception and design. Material preparation, data collection and analysis were performed by TB, ML, VS and EH. The first draft of the manuscript was written by TB and all authors commented on previous versions of the manuscript. All authors read and approved the final manuscript.

## FUNDING INFORMATION

This research received no specific grant from any funding agency in the public, commercial or not‐for‐profit sector.

## CONFLICT OF INTEREST

All authors declare no conflict of interest.

## Supporting information


Appendix S1

## Data Availability

Data available on request from the authors.
