# Supplementary material for: Exploring mental health clients' current medication knowledge, beliefs and experience with healthcare providers in the community in South Australia
Source: Health Soc Care Community. 2022 Sep 23;30(6):e5968–78. doi: 10.1111/hsc.14029 (PMC10086827; doi:10.1111/hsc.14029)
Supplement: Supplementary file 1 — Appendix S1 [file HSC-30-e5968-s001.docx]

**Appendices:**

**Appendix 1: Focus group session guide**

Questions for Interview with Client

Part I *(Broadly: Knowledge Based Questions)*

Medications: 4 major questions

1. When you first received your medication,
2. was it explained what the medication was for?
3. can you recall what the information was?
4. what written information did you receive?
5. What were you told about possible side effects from this medication and how these will be monitored?
6. Were you given advice on which side effect you should seek medical attention for?
7. Have you ever experienced any adverse effects?

Your health condition/s: 2 major questions

1. What health professionals are currently involved in your care?
2. Have you been offered other “non-pharmacological*” options alongside your medications for example psychological treatments?

*(*this may need explanation)*

If Yes to Q2, what are they?

Part II *(Broadly: Attitude or Perception Based Questions)*

Medications: 3 major questions

1. How do you feel about taking your medication?
2. In general, do you feel involved in decisions about your medications?
3. Do you ever miss a dose of your medication/s (either deliberately or inadvertently)?

If Yes to Q3, do you know what to do after missing a dose?

If Yes to Q3, are you comfortable speaking with your doctor or pharmacist about missed dose/s?

Your health condition/s: 3 major questions

1. What support could be offered by your doctor, pharmacist and other health professionals that you are not currently getting?
2. Can you recall a situation where you felt hesitant to speak with your doctor, pharmacist or other health professional about your health (or your medication) because you felt the query was not important enough?
3. On a scale of 1 – 5,
   1. how would you rate your experience with your doctor?
   2. how would you rate your experience with your pharmacist?
   3. how would you rate your experience with other health professionals?

This service: optional questions

1. What particular aspect of this service do you find useful?
2. What do you think could be done better to support you?
